# Advanced visual components inspired by animal eyes

**DOI:** 10.1515/nanoph-2024-0014

**Published:** 2024-03-01

**Authors:** Sehui Chang, Duk-Jo Kong, Young Min Song

**Affiliations:** School of Electrical Engineering and Computer Science, 65419 Gwangju Institute of Science and Technology (GIST), Gwangju 61005, Republic of Korea; Artificial Intelligence (AI) Graduate School, Gwangju Institute of Science and Technology (GIST), Gwangju 61005, Republic of Korea; Department of Semiconductor Engineering, 65419 Gwangju Institute of Science and Technology, Gwangju 61005, Republic of Korea

**Keywords:** bioinspired vision, optical design, nanostructures, image sensors, retinomorphic devices

## Abstract

Artificial vision systems pervade our daily lives as a foremost sensing apparatus in various digital technologies, from smartphones to autonomous cars and robotics. The broad range of applications for conventional vision systems requires facile adaptation under extreme and dynamic visual environments. However, these current needs have complicated individual visual components for high-quality image acquisition and processing, which indeed leads to a decline in efficiency in the overall system. Here, we review recent advancements in visual components for high-performance visual processing based on strategies of biological eyes that execute diverse imaging functionalities and sophisticated visual processes with simple and concise ocular structures. This review first covers the structures and functions of biological eyes (i.e., single-lens eyes and compound eyes), which contain micro-optic components and nanophotonic structures. After that, we focus on their inspirations in imaging optics/photonics, light-trapping and filtering components, and retinomorphic devices. We discuss the remaining challenges and notable biological structures waiting to be implemented.

## 1 Introduction

Vision is an important sensation of animals, allowing them to acquire vital information for the propagation of species in dynamically changing environments. Similarly, modern electronic devices mostly come equipped with a camera module, and sometimes more than one, to collect various visual information for different occasions. The fundamental configuration of digital cameras consists of front optics, including surface coatings, lens optics, and apertures, as well as photosensing units that feature complementary metal-oxide semiconductor (CMOS) sensors, often accompanied by optical filters. These visual components have been developed to capture optimal images containing essential information about the surroundings. For example, thin-film coatings are used on lens optics to minimize light loss through antireflection properties [1]. Lens design has diversified with the use of multiple lenses, aspheric and free-form surfaces, and combinations of different materials to achieve various optical features such as a wide field of view, zooming capability, and low aberration imaging [2]. Photosensors have evolved to have hundreds of millions of pixels with microscale sizes for high resolution images [3].

Nowadays, technological developments have transformed the camera from a standalone device into an artificial organ that performs visual processes to aid the decision-making of machines or robots. This transition necessitates tailored camera systems capable of providing specific imaging for target applications such as target-oriented imaging for drones [4], region of interest imaging for autonomous vehicles [5], and panoramic imaging for exploration rovers [6]. These systems conduct the entire visual process, from image collection to information extraction. Key requirements for artificial vision systems include light weight, low energy consumption, high compatibility with other electronics, and adaptability to environmental changes. However, limitations of bulky, complex, and single functional visual components in traditional camera systems impose the need for another technological breakthrough in the design, driving a renascence of bioinspired visual components.

Returning to natural vision systems, animal eyes exhibit unique structures and visual components optimized for their habitats. Fishes, for instance, observe panoramic visual fields underwater through their single ball lens systems, which compose a gradient refractive index profile, enabling wide field of view imaging with minimized aberrations [7]. Domestic cats and moths maximize incoming light during the nighttime using photonic structures with retroreflective and antireflective properties, respectively [8], [9]. Insects sensitively perceive moving objects through compound eyes consisting of thousands of micro-optics with photoreceptor cells [10]. Furthermore, the retinal signaling mechanism in human eyes demonstrates an economical visual process in terms of speed and energy consumption [11]. These environmentally adapted vision systems of animals, which exhibit remarkably simple but versatile ocular structures and a highly efficient visual processing scheme, have inspired many researchers to devise advanced visual components.

Here, we summarize recent advancements in visual components based on strategies found in biological eyes for highly efficient visual processing. With simple yet refined ocular structures and retinal processes, animals readily deal with visual information from their surroundings for survival, while minimizing time and energy consumption. In this review, we first broadly cover diverse ocular structures and functions of biological eyes optimized to dynamic visual environments. We then elaborate on advanced visual components in recent years inspired by animal eyes, encompassing surface structures, light-managing and focusing optics, light-trapping and filtering components, and retinomorphic devices. Simultaneously, representative visual components based on optical/photonic structural designs, micro/nano-fabrication, and optoelectronics are introduced with actual structures and functionalities in animal vision systems. We finally discuss remaining challenges in bioinspired visual components, major requirements for practical applications integrated with other devices, and notable biological structures waiting to be implemented in the future.

## 2 Structures and functions of biological eyes

Unlike most artificial vision systems based on the cameras consisting of a lens, aperture, and image sensor, biological eyes have diversified to fulfil the distinct demands of animals. Animal eyes can be mainly categorized in two divisions: camera-type eyes and compound eyes, which have evolved to improve spatial vision. It is somewhat unclear why biological eyes have evolved two different eye types during the improvement of spatial vision, but the diversity of structures and functions in animal eyes, even within the same eye type [12], has inspired researchers to develop artificial vision systems tailored to specific visual environments. For example, the camera-type eye, as its name suggests, has a lens, pupil, and retina, forming a configuration similar to that of cameras, providing high resolution compared to the compound eyes under the constraint of eye size [13]. However, the diverse living environments of animals with camera-type eyes result in distinctive ocular structures and additional visual functions, including a variety of pupil shapes, lens shapes and compositions, curved retinas, wide field of view, and spectral sensitivity. Meanwhile, compound eyes, commonly found in insects and crustaceans, exhibit unique array structures composed of several or thousands of optical elements, providing a wide field of view, high motion sensitivity, and infinite depth of field [14]. The variations of compound eyes among species imply multifarious optical features, such as amphibious vision, high sensitivity, and multispectral vision. Hence, these diverse eye configurations enable animals to extract essential visual information from their surroundings for mating, predatory, and vigilance behaviors, guiding researchers to develop advanced visual components by mimicking their eyes.

### 2.1 Camera-type eye systems

**Figure 1:**
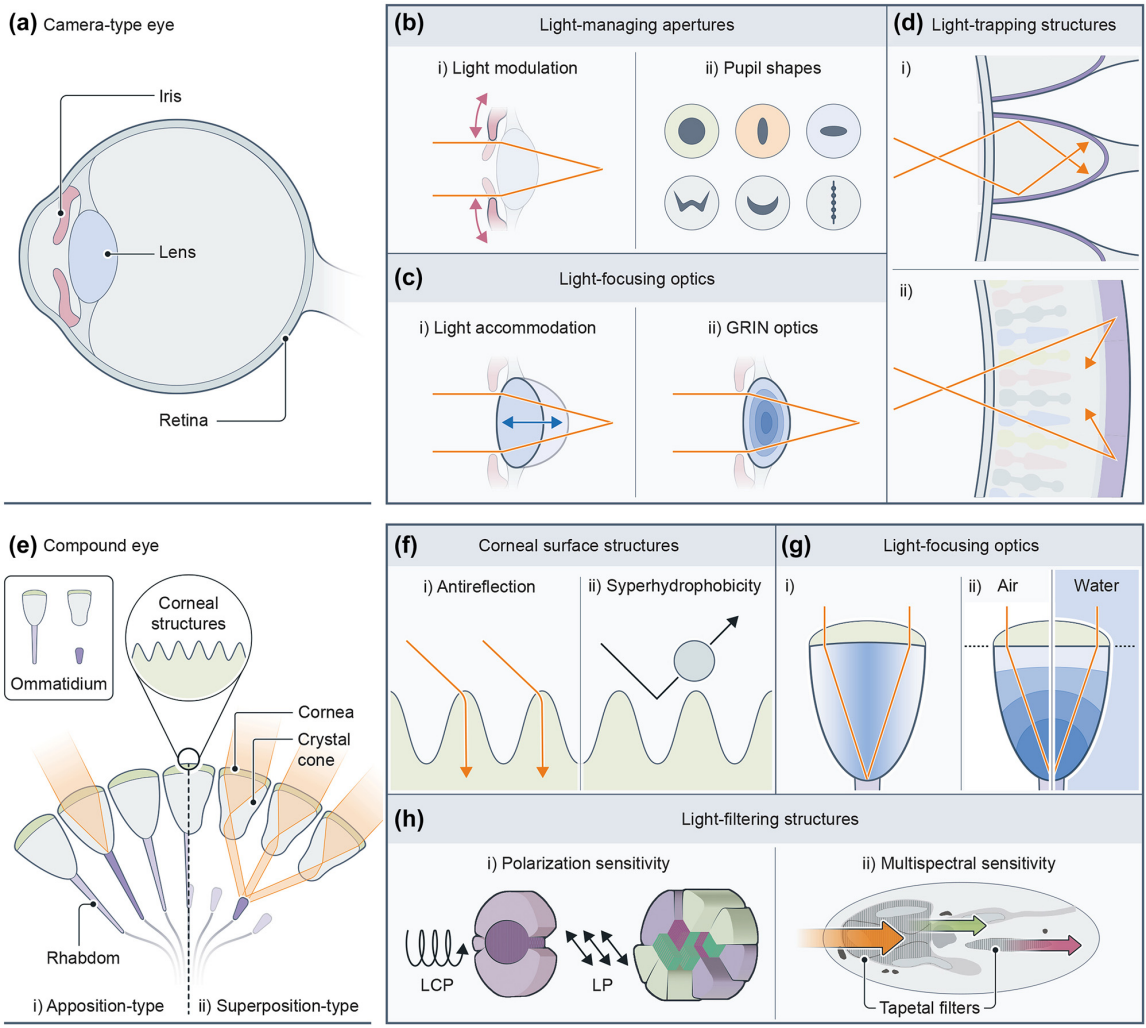
Biological eye structures and major optical/photonic components. (a) Camera-type eye. (b) Light-managing apertures. (c) Light-focusing optics. (d) Light-trapping structures. (e) Compound eye. (f) Corneal surface structures. (g) Light-focusing optics. (h) Light-filtering structures.

The most representative example of camera-type eyes would be the human eye, which leads to the development of the cameras. However, camera-type eye systems can be found in many vertebrates and invertebrates. As mentioned, the visual components of camera-type eyes typically include the pupil, which modulates the amount of light approaching the retina by adjusting its area (or size); the lens, which focuses images on the retina through variations in its shape and refractive index distribution; and the retina, which absorbs and converts focused light into electrical signals through photoreceptors (Figure 1(a)).

The diverse environments give rise to variations in visual components among species, adapting to their ecological niches. First, the pupil primarily plays a role in modulating the amount of incoming light by changing its size or area via constriction or dilation movements of ciliary muscles, responding the intensity of illumination, which is related to the sensitivity of the eye (Figure 1(b)(i)). For instance, the circular pupil of the human eye changes its opening area by approximately 15 times, while the vertical slit-like pupil and vertically beaded pupil of the domestic cat and Tokay gecko change by approximately 135 and 350 times, respectively [15]. However, the various pupil shapes (e.g., round, slit-like, and irregular shapes) imply additional functions beyond light modulation (Figure 1(b)(ii)). For example, vertical slit-like pupils are hypothesized to enhance depth estimation for ambush predators, including felines; and the horizontal slit-like pupils found in goats, reindeers, and horses are suitable for prey animals that need a horizontally wide field of view [15]. Moreover, many species exhibit irregular-shaped pupils including W-shaped [16], crescent (e.g., U-shaped) [17], beaded (i.e., multiple pinholes in a row) [18], heart-shaped [19], rhomboidal [20], and fan-shaped (or inverted fan-shaped) [19] pupils. They are known to provide unique optical features, which correlate specific needs for each species including habitats and foraging behaviors, such as balancing uneven illumination in the W-shaped pupil of cuttlefish [16], correcting chromatic aberrations in the crescent pupil of some cephalopods [17], estimating depth with monocular vision in the beaded pupil of geckos [18].

Next, the camera-type eye focuses images onto the retina through the crystalline lens. These lens optics vary among animals, exhibiting different focusing abilities such as shapes of the lens surface, refractive indices of the lens, and the lens configuration. For example, the lens of the human eye is sophisticatedly adjusted in thickness to clearly focus on objects near and far (Figure 1(c)(i)) [21]. Aquatic animals like fish have a gradient refractive index profile on their ball lens, which refracts incoming light with minimized optical aberrations (Figure 1(c)(ii)) and accommodates to focus on near and far objects by modulating distances from the rear lens surface to the retina [22]. Furthermore, several animals possess multiple lens configurations similar to manmade lens design (e.g., doublet and triplet), as in the ventral eye of a male *Pontella* forming the triplet lens with a parabolic surface that can provide a sharp focus even without a gradient refractive index [9].

Lastly, structures and functions of the retina in camera-type eyes are also diverse across species, ranging from curved geometry and/or photoreceptor cell density distribution to photonic structures of the retina. In the eye of the elephantnose fish, photonic crystal cup structures have been found in the retina, which can enhance light collection in specific wavelengths by working as a reflecting sidewall enabling to achieve more sensitive vision in the dim and turbid water (Figure 1(d)(i)) [23]. As another strategy for high sensitivity, tapetum lucidum, are commonplace mirror-like photonic crystal structures found in the retina of vertebrates and arthropods [24]. In some cases of nocturnal animals, the tapetum lucidum positioned behind the photoreceptor cells offer a second chance to absorb transmitted light by reflecting it back to the photoreceptors as a retroreflector, thereby enhancing the overall retinal sensitivity (Figure 1(d)(ii)) [25].

### 2.2 Compound eye systems

Although the camera-type eye is the most familiar owing to its association with the human eye, compound eyes are more prevalent eye type in nature, found in a wide range of species including insects, as commonly known, and crustaceans. Unlike the singular optics system in the camera-type eye, a hemispherical array of hundreds or thousands of optical units, called ommatidia, constructs monocular vision (Figure 1(e)). In particular, compound eyes can be divided into two types: apposition-type in diurnal insects (e.g., dragonflies and bees) (Figure 1(e)(i)) and superposition-type in nocturnal insects (e.g., moths) and deep-sea crustaceans (e.g., lobsters and crayfish) (Figure 1(e)(ii)), implying variations in eye structures. Consequently, the visual components in compound eyes can be categorized into two major parts: micro-optics, including micro- or/and nano-structures, microlenses, and crystal cone; and rhabdoms, including photoreceptor cells, and in some cases, light filtering structures.

Moths and mosquitoes possess nano- or/and micro-scale nipple arrays on the corneal surface of their eyes (Figure 1(f)). Notably, these miniscule surface structures provide broadband antireflection properties via moderate changes between air and lens facet, resulting in minimized light loss and enhanced sensitivity [26], while achieving superhydrophobicity under foggy or highly humid conditions [27]. As found in the lenses of camera-type eyes, crystal cones with gradient refractive index profile are also present in the eyes of Antarctic krill, classified as the superposition-type (Figure 1(g)(i)) [28]. The refracted light beams from adjacent crystal cones are superimposed to achieve improved light intensity. Another example of ecological adaptation in lens optics can be found in fiddler crabs, which live in the intertidal zone. The layered lens with a flat surface on top enables them to focus light regardless of refractive index changes (e.g., air and water) in outer media (Figure 1(g)(ii)) [29].

The light focused from the anterior micro-optics is filtered to extract spectral or/and polarization information in some species, and then absorbed photoreceptor cells. For example, some beetles and mantis shrimps are known that they recognize circularly polarized light reflected from their conspecifics [30], [31]. The directionally aligned microvilli stacks in the ommatidia absorb or transmit incoming polarized light, with the orientation of microvilli alignment determining the direction of the detected polarized light (Figure 1(h)(i)) [32], [33]. Meanwhile, photonic crystal structures for multispectral vision are found in the ommatidia of butterfly eyes (Figure 1(h)(ii)). The tapetal filter layers are stacked with air layers alternatively, resulting in multispectral sensitivity throughout the ultraviolet (UV), visible, and near-infrared (NIR) wavelength region, as well as the eye shine [34].

## 3 Bioinspired visual components for artificial vision systems

Animal eyes that evolved to adapt to ecological niches have achieved high performance vision with relatively simple, small, and economic visual systems. These refined design strategies of biological eyes have been explored over the past few decades to develop advanced visual components (e.g., optical coatings, lenses, filters, sensors, and processing units) beyond the conventional manmade camera systems. Progress in imaging technologies enables meticulous observation of ocular structures in nature, while advancements in nanofabrication and optoelectronic technologies allow the development of bioinspired visual components. These trends have attracted many researchers in the field to implement artificial vision systems for high quality and energy-efficient visual processes.

### 3.1 Surface structures inspired by animal eyes

**Figure 2:**
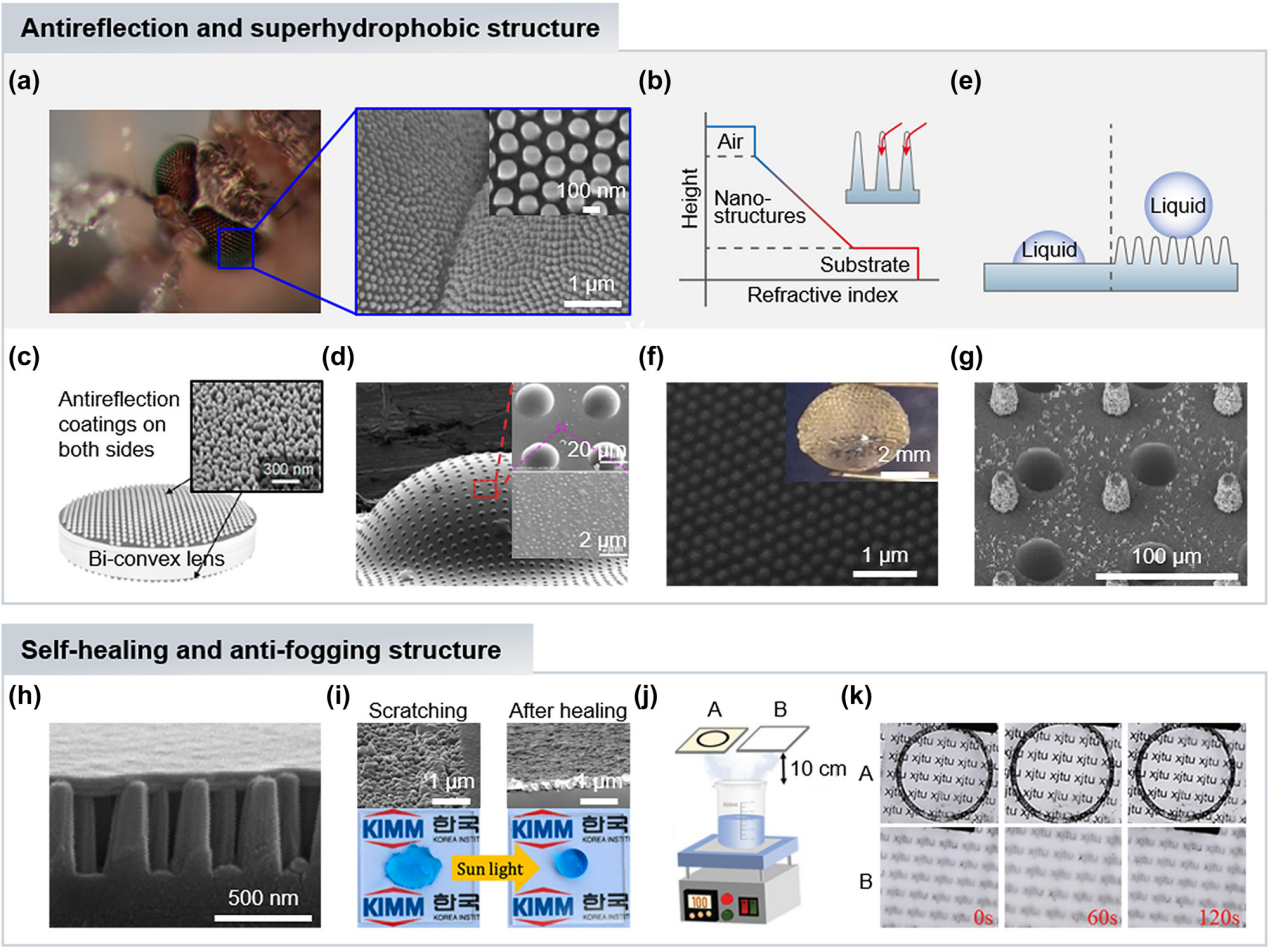
Bioinspired antireflection and superhydrophobic structures. (a) Antireflective nanostructures of mosquito’s eyes (left) and scanning electron microscope (SEM) images of enlarged view (right and inset). Reproduced with permission [35]. Copyright 2007, Wiley-VCH GmbH. (b) The refractive index change along the height of nanostructures from air to substrate. (inset) Schematic illustration of light passing through tapered nanostructures as the refractive index changes gradually. (c) Schematic illustration of biconvex lens with antireflection coatings on both sides. The inset is SEM image of fabricated SiO_2_ nanostructures. Reproduced with permission [36]. Copyright 2019, MDPI. (d) SEM images of artificial compound eye with micro/nanostructures. The insets are enlarged views of microlenses (top) and micro/nanostructures (bottom). Reproduced with permission [42]. Copyright 2021, American Chemical Society. (e) Schematic illustration of water droplets on a flat (left) and nanostructured (right) surfaces. (f) SEM image of silica nanoparticles hexagonally arranged on the microlens surfaces. The inset is SEM image of fabricated convex-convex type compound lens with nanostructures on the microlenses. Reproduced with permission [47]. Copyright 2019, American Chemical Society. (g) SEM images of microlenses with hierarchical pillar arrays. Reproduced with permission [48]. Copyright 2021, American Chemical Society. (h) SEM image in cross-section view of bioinspired self-healing nanostructures with paraffin coating layer. (i) SEM images of scratched (left top) and self-healed (right top) structures. Self-healing properties through sunlight treatment by testing hydrophobicity (left bottom) after the mechanical damages and (right bottom) after the sunlight treatment. Reproduced with permission [49]. Copyright 2020, American Chemical Society. (j) Schematic illustration of anti-fog experiment setup with anti-fog structured sample A and bare glass sample B. (k) The result optical images in anti-fog experiment of sample A and B. Reproduced with permission [50]. Copyright 2023, Wiley-VCH GmbH.

Antireflective (AR) coatings play a crucial role in optical science for minimizing light loss due to surface reflection. Typically, the thin-film-based approach is a well-known AR coating technique that utilizes destructive interference of two different interfacial reflections in a dielectric thin-film on a substrate [1]. Despite their simplicity in fabrication, limitations in narrow bandwidth, incident angles and polarization have led to the exploration of alternative AR coating methods. Meanwhile, various AR structures exist in nature, from butterfly wings and bird feathers to plant leaves. In insect compound eyes, micro- or/and nano-structures such as the nanonipple array in the corneal surface of mosquito eyes (Figure 2(a)), serve as AR coatings, increasing light sensitivity in dim environments [35]. The AR property is achieved as light travels onto nipple structures exhibiting tapered morphologies on a sub-wavelength scale, which suppresses light reflections and increases transmittance. Since light is not affected by individual nipples owing to their sub-wavelength scale, it instead undergoes a gradual refractive index change from air to substrate (Figure 2(b)). This reduces reflectance generated by sudden refractive index changes at the interface between two different media, allowing for broadband, omnidirectional, and polarization-insensitive AR coatings, outperforming conventional methods.

Among various visual components, lens optics, in particular, face challenges with surface reflections leading to light loss and glare in images. Researchers have explored biomimetic AR structures for optical lenses to increase light intensity and minimize reflections. For instance, a biconvex lens AR coated on both sides mimics corneal nipples in a moth’s eye (Figure 2(c)) [36]. The design considered parameters such as height, period, and morphology (e.g., rods, truncated cones, and cones) to optimize AR properties, otherwise light scattering occurs, reducing surface transparency. Simulation results based on the rigorous coupled-wave analysis (RCWA) method showed that truncated-cone nanostructures have better transmittance across a broad wavelength range from 400 to 1600 nm. Also, the period and height were optimized to 200 nm and 150 nm, respectively, and the transmittance increases as a filling fraction increases. The designed nanostructure was fabricated on the curved surface of a biconvex lens through top-down methods of dry etching the deposited SiO_2_ using thermally dewetted Ag as a hard mask. Compared to bare lenses, AR coated SF11 and BK7 lenses showed increased transmittance at broadband wavelengths.

In general, the fabrication of AR structures includes a patterning process to transfer desired nanopatterns onto the target substrate via lithography techniques, and an etching process to form designed nanostructures. For transferring sub-wavelength patterns, various lithography techniques are adopted, such as photolithography based on photosensitive materials, colloidal lithography based on the monolayer of nanospheres [37], and nanoimprint lithography based on the prepatterned master mold [38]. Then, the etching process is carried out to develop patterned structures via dry or wet etching techniques. For example, Taniguchi’s group reported a micro-lens array with AR structures via nanoimprint lithography and multiple replica molding processes [39]. A flexible replica mold with AR moth-eye nanostructures successfully transferred its reverse pattern onto the concave microlens array mold, so that the fabricated microlens array has AR structures on the surface. Meanwhile, Gao’s group fabricated AR nanostructures via colloidal lithography and a dry etching process [37]. The self-assembled polystyrene (PS) monolayer acts as a mask for nanocone arrays during the reactive ion etching process, and the AR structures can be optimized by tuning the size of the PS nanosphere.

Recent advancements in elastomeric materials have driven significant attention to developing flexible optical systems such as tunable lenses, which can adjust focal length by changing lens shapes with fewer lenses compared to traditional rigid glass lenses. For the tunable lens, AR coatings are also important to reduce reflections and increase image brightness. However, it is quite challenging to realize AR properties on the tunable lens with conventional thin-film methods because of their rigid nature. In this regard, bioinspired AR structures on a flexible substrate have been demonstrated [40], [41]. For example, the tunable and antireflective endoscopic lens has been demonstrated by constructing bioinspired AR nanostructures on a highly flexible polymeric lens membrane of a tunable liquid-filled lens for endoscopic laparoscopy [40]. The nanohole template was built though a solid-state dewetting process of a thin silver film, and then, AR structural patterns were transferred by replica molding a polydimethylsiloxane (PDMS) lens membrane. The fabricated AR PDMS membrane presented improved transmittance of 96.8 % and 95.5 % in flat and curved states, respectively, enhancing the image contrast of the final AR tunable endoscopic lens.

To exploit the optical advantages of compound eyes, artificial compound eyes (ACEs) with AR structures have been demonstrated using various fabrication methods such as direct laser writing, soft lithography, and nanoimprinting processes. Yin’s group demonstrated hierarchical AR structures on the artificial compound eyes via nanotip-focused electrohydrodynamic jet (NFEJ) printing techniques (Figure 2(d)) [42]. A solid Ni nanotip utilized in the NFEJ printing allows solving blockage issues in the nozzle when using high viscosity materials, so that the UV curable polymer can be directly printed on the PDMS film substrate to form a microlens array and micro/nanostructures, reducing complex manufacturing steps in conventional methods. In addition, the micro/nanostructures decrease light reflectance more effectively than the others without structures and only with nanostructures. The flat ACEs were deformed into the curved shape like insect eyes through pneumatic methods owing to the flexibility of the PDMS film.

Interestingly, corneal nipple arrays in moth’s and fly’s eyes offer another function of self-cleaning/anti-fogging due to the dewetting properties of nanostructures [43], [44]. Unlike smooth surfaces that are rather difficult to repel water droplets without chemical hydrophobicity on their surface, rough surfaces can exhibit hydrophobicity with a high contact angle of the water droplet beyond 120° (Figure 2(e)). In general, the wettability on rough surfaces can be characterized by two models of Wenzel and Cassie–Baxter states. While the Wenzel state has a two-phase of liquid–solid interface in which the droplet surface directly contacts the rough surface, air pockets exist between the droplet surface and rough structures in the Cassie–Baxter state, exhibiting superhydrophobic features [45], [46].

Inspired by mosquito’s eyes, the ACE with multifunctional hierarchical structures that present a wide field of view, infinite depth of field, and antifogging characteristics have been developed (Figure 2(f)) [47]. The assembly of liquid marbles, emulsion droplets, and nanoparticles successfully mimicked the hierarchical structures and their multiple functionalities. The photocuarable oil droplets were polymerized and collected to be used for a microlens array via capillary assembly, and then decorated with silica nanoparticles for antifogging properties, showing superhydrophobicity with a contact angle of 165°. Another ACE was fabricated through the incorporation of photolithography, inkjet printing, and chemical growth, which presents superhydrophobic features while maintaining the optical performance with high transparency (Figure 2(g)) [48]. The nanohairs are chemically grown on the fabricated microcone arrays, forming hierarchical structures. This hierarchical morphology provides stable superhydrophobicity with a contact angle of 160°, endowing great performance under both static and dynamic wetting states.

Mechanical robustness is an essential feature for the practical application of AR structures. Recently, self-healing structures with AR properties have been demonstrated using moth-eye-inspired nanopillar structures with paraffin layer coatings (Figure 2(h)) [49]. Although scratching degrades superhydrophobicity of the structures by damaging paraffin coatings, they can recover the hydrophobicity through sunlight treatment (Figure 2(i)), even after repeated damages, providing an alternative to enhance the durability of AR structures. Meanwhile, mammalian eyes have a great antifogging mechanism preventing image degradation in humid conditions [50]. The cornea of warm-blooded animals, including humans, shows high resistance to fogging through thermoregulation by maintaining the eye’s temperature to exceed that of the surroundings, and tears, which keep the cornea consistently wet. This natural mechanism inspires the development of antifogging slippery glass surfaces [50]. The femtosecond laser wet etching process was employed to create an empty cabin within silica glass. Subsequently, the cabin is filled with graphene to facilitate heat transfer from sunlight, effectively hindering fog generation (Figure 2(j) and (k)). To enhance durability and enable self-healing, a shape memory polymer to encapsulate the graphene and additional surface treatments to achieve slippery and self-healing properties were adopted.

### 3.2 Apertures and focusing optics inspired by animal eyes

**Figure 3:**
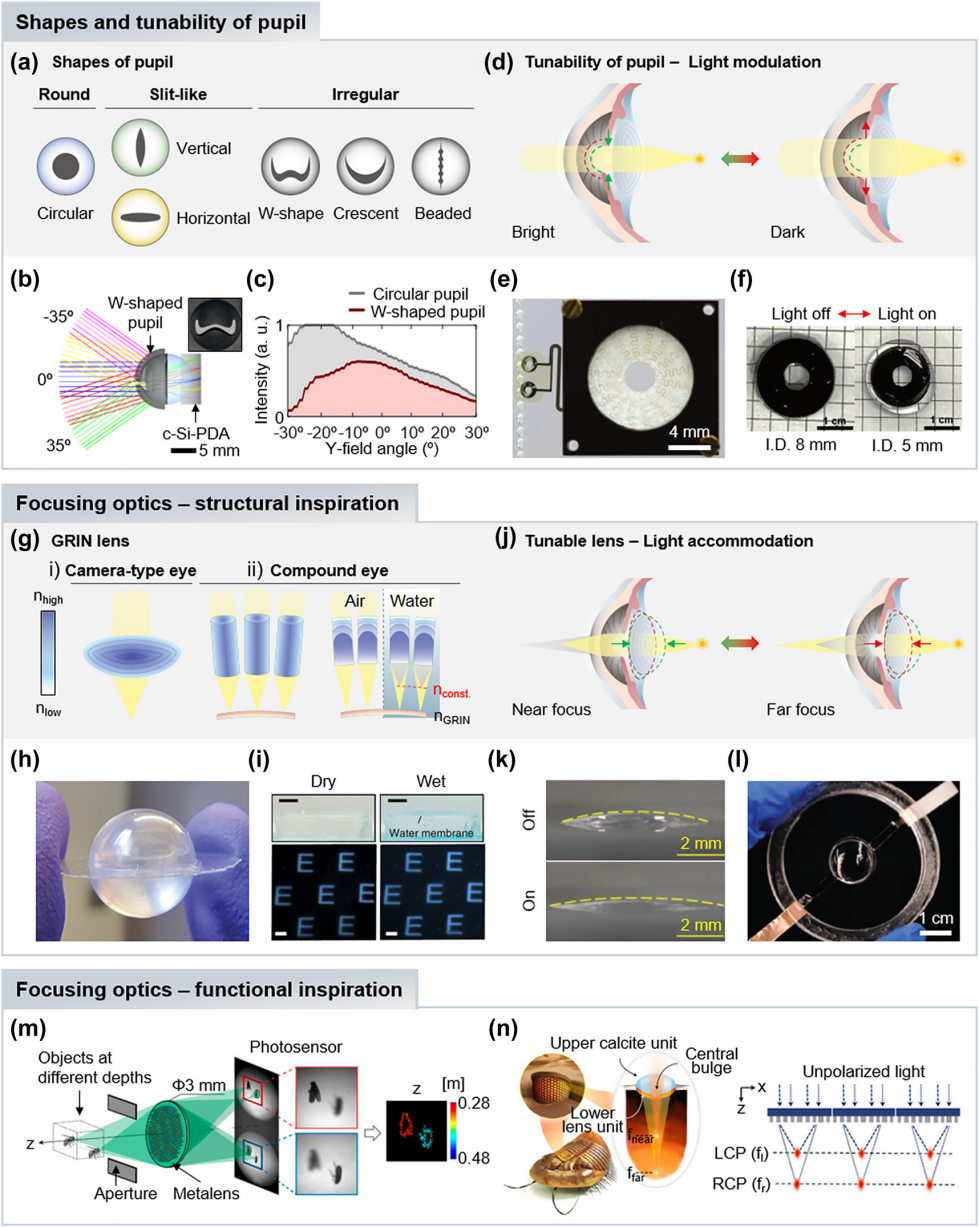
Bioinspired optical components. (a) Diverse pupil shapes in biological eyes. (b) Optical layout of cuttlefish-inspired lens with W-shaped pupil. The inset is fabricated W-shaped aperture. (c) Light balancing performance of circular and W-shaped pupil imaging systems. Reproduced with permission [5]. Copyright 2023, American Association for the Advancement of Science. (d) Light modulations by adjusting the pupil size. (e) The liquid crystal elastomer-based tunable iris with embedded heaters. Reproduced with permission [54]. Copyright 2016, Springer Nature. (f) Tunable iris based on NIR-sensitive hydrogels. Reproduced with permission [55]. Copyright 2022, American Chemical Society. (g) Schematic illustrations of GRIN lenses in camera-type and compound eyes. (h) Image of a GRIN ball lens consisting of two hemispherical GRIN lenses. Reproduced with permission [56]. Copyright 2013, SPIE. (i) Focusing performance of graded microlenses under dry and wet conditions. Reproduced with permission [58]. Copyright 2022, Springer Nature. (j) Light accommodations by modulating lens shape. (k) Different shapes of the inkjet printed gel lens in ‘On’ and ‘Off’ states at rest and with applied voltage. Reproduced with permission [62]. Copyright 2020, Wiley-VCH GmbH. (l) Image of a PEE-based tunable lens. Reproduced with permission [63]. Copyright 2022, Wiley-VCH GmbH. (m) Depth estimation of two objects at different distances through the metalens depth sensor inspired by the jumping spider. Two images that focus on different objects provide the computed depth map on the right. Reproduced with permission [70]. Copyright 2019, National Academy of Sciences. (n) (right) Schematic illustration of a trilobite and its ocular structure. (left) Schematic illustration of the photonic spin-multiplexed metalens array that has two focal planes of LCP and RCP light. Reproduced with permission [71]. Copyright 2022, Springer Nature.

Pupils in animal eyes regulate the amount of light reaching the retina, responding dynamically to ambient light intensity. The size of the opening area in pupils affects field angles and the beam size of light incidence. Animals exhibit different pupil shapes such as round, slit-like, and irregular types, varying among species and their habitats (Figure 3(a)). For example, felines have vertical slit-like pupils specialized in depth estimation, leveraging the focusing difference between vertical and horizontal contours along the depth. Indeed, vertical slit-like pupils are advantageous for ambush predators, accurately estimating prey distances, while horizontal slit-like pupils facilitate facile detection of predators through a horizontally wide field of view [15]. Irregular types, such as W-shaped, crescent, and beaded pupils, exhibit unique optical characteristics like light balancing [16], chromatic aberration correction [17], and depth estimation in monocular vision [18]. While modern camera systems predominantly use circular apertures imitating the human eye, researchers draw inspiration from various pupil shapes to develop tailored apertures for specific imaging applications.

Artificial camera systems with a W-shaped pupil, inspired by cuttlefish (genus *Sepia*), have been recently demonstrated as a potential candidate for autonomous vehicles during the daytime [5]. Cuttlefish living in shallow water constrict their pupils into a W-shape, addressing the challenges posed by vertically uneven illumination due to sunlight in the sky. Inspired by the cuttlefish vision, Song’s group designed artificial vision systems comprising a W-shaped aperture, balls lens, and cylindrically curved photodiode array for autonomous vehicle cameras, which encounter similar light environment from the intense sunlight in daytime (Figure 3(b)). Optical simulations and imaging demonstrations revealed that the W-shaped pupil effectively balances vertical illumination by reducing intense light from upper sides (Figure 3(c)).

In dynamic ambient light environments, light modulation is crucial for animals to avoid light saturation or a deficiency of light. The pupil adjusts its opening area according to ambient light levels, representing a rudimentary light adaptation (Figure 3(d)). Similarly, camera systems require dynamically tunable apertures to collect high contrast images. In recent years, various tunable apertures have been developed by mimicking the light modulation of pupils in natural eyes [51], [52], [53]. In 2016, Zappe’s group introduced a tunable imaging system utilizing soft-matter-based optical components, including a tunable lens and iris [54]. The tunable iris, fabricated by using a liquid crystal elastomer (LCE), changes its radial contraction using embedded heaters (Figure 3(e)). Another tunable iris, photosensitive to NIR light, has been reported [53]. The artificial iris was fabricated by thermally reacting LCEs, so that the elongation and contraction of LCEs are triggered by thermal actuation. Then, they coated the surface of the fabricated LCE iris with photosensitive materials, polydopamine (PDA), for photoactuating its shapes. The PDA-coated LCE iris presents reversibility according to NIR light exposure. Biocompatibility is frequently required for visual components when used in biomedical applications such as prostheses and endoscopy. However, the compatibility of LCEs with biological eyes is quite low to directly utilize the artificial LCE iris. To resolve challenges of biocompatibility in LCEs, Chen’s group adopted hydrogels, which are actively used in biomedical devices, and demonstrated a hydrogel-based tunable iris [55]. The synthesized double-layered hydrogel responds to temperature changes, exhibiting thermal actuation. In order to endow photosensitivity, the PDA was coated to activate thermal actuation of the hydrogel iris by converting NIR energy to thermal energy. The fabricated PDA-coated hydrogel iris exhibited reversible actuation triggered by NIR irradiation (Figure 3(f)).

The primary visual component responsible for focusing images is lens optics. Various lens parameters including surface shapes, refractive index of materials, and lens combinations, involve image quality and contribute to advancements in lens design. Conventional camera systems typically select multiple lens configurations to focus images on flat sensors with high imaging performance. However, this complex design comes with limitations in size reduction, precise alignment, and manufacturing cost, which are unfavorable for compact cameras to be utilized in robotics and mobile applications.

In contrast, animal eyes efficiently perform visual processes needed for survival with relatively simple and tiny focusing optics, typically comprising one or few lenses. Single lens systems in natural eyes often feature a gradient index (GRIN) profile (Figure 3(g), left). The homogenous index lens induces severe spherical aberrations due to different focal points between on- and off-axis rays, requiring additional lenses or aspheric surfaces for correction. On the other hand, the fish eye focuses high quality images with a single ball lens that presents a parabolic GRIN profile, remarkably eliminating spherical aberrations [7]. Inspired by the biological lens, the hemispherical GRIN lens was fabricated by nanolayering polymer films [56]. The tailoring of the refractive index was conducted by controlling the volumetric contribution of two polymers, PMMA and SAN17. To fabricate ball lens with GRIN film stacks, three GRIN shells with different diameters constructed by diamond tuning of film stacks were assembled with a SAN 17 ball lens (Figure 3(h)).

The GRIN profile is also found in the compound eyes of krill and fiddler crabs (Figure 3(g), right). For example, Raskar’s group integrated wide angle imaging systems inspired by the superposition type compound eyes of krill [57]. The GRIN lenses are aligned on a curved surface to provide an undistorted, uniform image for rotation angle estimation, showing potentials for wide angle cameras. The fiddle crab, living in intertidal zone, focuses clear images in both land and water. This is because of the layered structural corneal lens with a flat surface on top, which results in a GRIN profile achieving constant focal lengths regardless of the outer media (e.g., air and water) [29]. Recently, artificial compound eye systems that can provide amphibious and panoramic vision have been demonstrated by mimicking fiddler crab eyes [58]. They fabricated a flat microlens array by stacking optical layers with different refractive indices to focus in both dry and wet conditions (Figure 3(i)), suggesting that the flat GRIN lens can be utilized for amphibious imaging without additional lenses.

In conventional camera systems, focus adjustment is achieved by precisely shifting the lens along the optical axis using motors, resulting in bulky and slow system configurations. On the contrary, natural eyes easily handle light accommodation for focus adjustments between near and far objects, by deforming the lens shape (Figure 3(j)). In the human eye, the constriction or relaxation of ciliary muscles controls lens curvature, thereby changing the focusing power of the lens. The relaxation of the ciliary muscles decreases the radii of the lens curvature, increasing optical power. This adjustment enables near focus, and the process also operates vice versa. To achieve facile accommodation, the development of tunable optics has garnered interests in recent years [59], [60], [61].

The tunable lens based on transparent and conductive gels have been demonstrated for improving in compactness, fast responsivity, and extended focal length range [62]. A plano-convex gel lens was fabricated through inkjet 3D printing, enabling the precisely patterning of lenses with desired curvatures. Notably, the conductive gel operates itself as actuating electrodes, inducing Maxwell force to stretch the gel lens in radial direction (Figure 3(k)), while exhibiting high transparency. The tunable gel lens attains a relatively large variation in focal length of 32–81 % and millisecond-scale fast response. Another electrically tunable lens was developed by mimicking the light accommodations of human eyes [63]. The conductive polyelectrolyte elastomer, poly(3-acylamidopropyl) trimethylammonium chloride, was used as both actuating electrodes and tunable lens regions (Figure 3(l)). The focal length can vary up to 46.4 % of a relative change at 9 kV of applied voltage, which is greater than that of human eyes.

While traditional lens optics, including rigid glass or/and plastic lenses, and flexible polymer lenses, continue to progress in achieving thin and compact lens systems, metalenses have emerged as another strategy to produce unconventional thin and planar optical components. Conventional refractive lens optics correct optical aberrations based on combinations of lens materials with different refractive indices and multiple surface designs, which incur challenges in further miniaturization and increase complexity in manufacturing. In contrast, metasurfaces can correct optical aberrations, including chromatic aberrations or spherical aberrations, by customizing an effective refractive index and dispersion to manipulate the light path through the design of geometrical parameters and the arrangement of subwavelength nanostructures [64], [65]. Besides, these nanostructures exhibit uniform height, benefiting compactness through their flat surface. The advantage of the wavefront shaping scheme in metalenses also facilitates research efforts in bioinspired optical components to realize various optical functions of animal eyes [66], [67], [68].

Wide field of view is one of the desirable optical properties in focusing optics. Many lens designers, until today, have delved into panoramic lens designs called fisheye lenses to achieve an extremely wide field of view of around 180°, similar to that of fish eyes in nature. However, fisheye lens design requires a relatively large diameter for the first lens component, with sophisticated designs in the rest of the lenses to compensate for aberrations and integrate with planar image sensors [69]. In this regard, Kim’s group presented wide field of view artificial eye systems composed of a single monocentric ball lens and curved photodiode array inspired by the actual ocular structures of fish [7]. As another strategy, a fisheye metalens that have a wide field of view near 180°, which far exceed the performance of single-layer metalenses, have been demonstrated by utilizing Huygens meta-atoms [66]. The design configuration of a front aperture stop and the metasurface on the backside of the CaF_2_ substrate enables different incident field angles passing though the aperture stop to reach the metasurfaces in a different but continuous manner. Moreover, by correcting aberrations such as coma, astigmatism, and, especially field curvature, the fisheye metalens can be compatible with commercial flat image sensors.

Zickler’s group demonstrated the metalens-based depth sensors by imitating the depth perception mechanism of jumping spiders [70]. The retina of jumping spiders constitutes a layered configuration, which enables the estimation of the depth of distant objects using degrees of blur on images. Instead of the use of multiple image sensors, they designed the metalens to focus two different object planes by forming two separate images on the sensor simultaneously (Figure 3(m)). Therefore, every shot produces two images focused on different depth objects, and subsequently, the depth map can be computed from defocus data.

Another group developed a nanophotonic light-field camera inspired by the compound eye of trilobites, achieving large depth of field range [71]. The lens optics of trilobites allow them to focus on near and far objects in the central bulge area of the upper calcite and lower lens units, respectively. This inspiration leads to the metalens design having two focal planes of circularly polarized light based on a spin-multiplexing approach (Figure 3(n)). With the incorporation of a reconstruction algorithm, the nanophotonic light-field camera successfully collected high quality light-field images over extremely large depth of fields, as well as low optical aberrations.

### 3.3 Light-trapping components and optical filters inspired by animal eyes

The diverse visual environments among species lead to the evolution of unique ocular structures for efficient visual processes. Together with the previously mentioned surface structures and lens optics, animals have sophisticated their retinal structures to collect high contrast images. In order to maximize light intensity in dim conditions, various nocturnal animal eyes feature photonic crystal structures in the retina for light-trapping, such as mirror-like cups and tapetum lucidum. For specific needs, several species (e.g., mantis shrimp, cephalopods, and butterflies) have nanostructures to recognize polarized light reflected from the fin of the prey and multispectral vision for discriminating conspecifics. Recent advancements in photonics and nanofabrication technologies have laid the foundation for developing light-trapping components and optical filters (e.g., polarization- or multispectral-sensitive) by exploiting remarkable strategies found in animal eyes.

**Figure 4:**
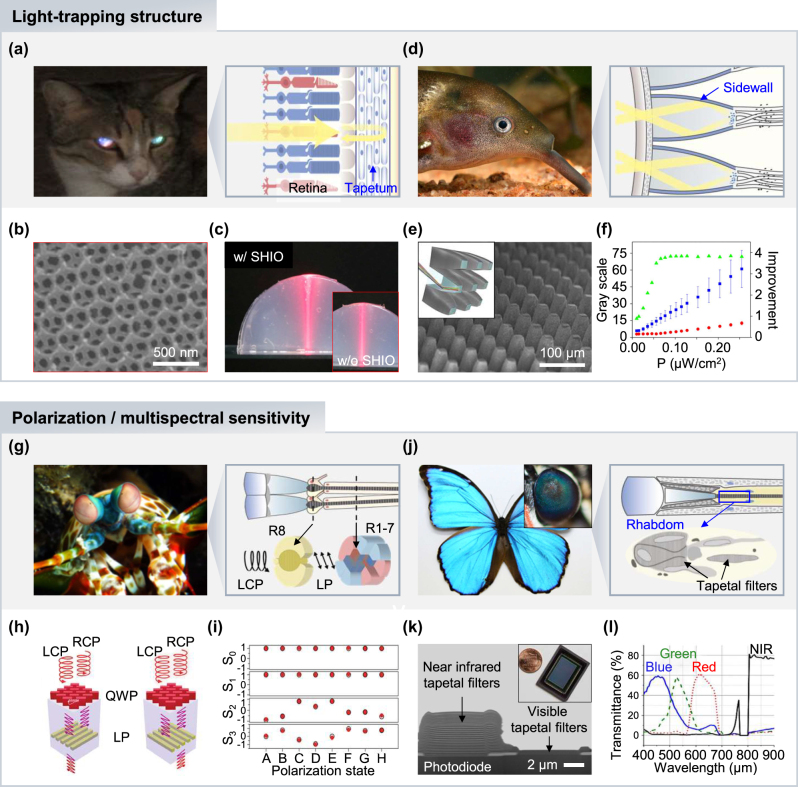
Bioinspired light-trapping structures and polarization/multispectral sensitive devices. (a) Photo of a cat with shiny eyes at dim light condition and schematic illustration of its retina anatomy. Reproduced with permission [8]. Copyright 2022, Wiley-VCH GmbH. (b) SEM image of SHIO. (c) Photos of reflection performance in the hemispherical gel with and without (inset) SHIO. Reproduced with permission [75]. Copyright 2017, National Academy of Sciences. (d) Photo of the elephantnose fish “*Gnathonemus petersii*” and schematic illustration of its retina anatomy. Reproduced with permission [125]. Copyright 2014, Wiley-VCH GmbH. (e) SEM image of BPE on a hemispherical substrate and schematic illustration of microphotocollectors (inset). (f) Light intensity enhancement along different light power intensities (green triangles). Blue squares and red dots are gray scale values collected by with and without BPE, respectively. Reproduced with permission [76]. Copyright 2016, National Academy of Sciences. (g) Photo of a mantis shrimp and schematic illustration of its retina anatomy. Reproduced with permission [126]. Copyright 2021, Wiley-VCH GmbH. (h) Schematics of stacked metasurface layers acting as a stack of R8 and R1–7 cells in mantis shrimp’s ommatidia. (i) Stokes parameters (*S*_0_–*S*_3_) according to eight random input polarization states. Black circles are results using a polarization analyzer and red circles are from the fabricated device. Reproduced with permission [80]. Copyright 2019, Springer Nature. (j) Photos of the Morpho butterfly and its eye (inset), and schematic illustration of its retina anatomy. Reproduced with permission [127]. Copyright 2019, American Chemical Society. (k) SEM image of fabricated tapetal filters on the image sensor. The inset is a photo of bioinspired multispectral image sensor. (l) Transmittance of the four different tapetal spectral filters of blue, green, red, and NIR channels. Reproduced with permission [97]. Copyright 2018, Optica Publishing Group.

Fishes and some terrestrial animals possess photonic crystal structures called tapetum lucidum, positioned behind the photoreceptor cells (Figure 4(a)). The tapetum lucidum reflects light back to the same direction of incidence (i.e., retroreflection), allowing an extra chance for photon captures of transmitted light, enhancing overall photosensitivity in dim conditions. This retroreflective characteristic is also responsible for the eye shine in some species, such as domestic cats [25], [72] and reindeers [73]. The retroreflective features of tapetum lucida can be utilized on the image sensor for improving photon catch of each photodiode, similar to the mechanism in animal eyes. Recently, several photonic crystal structures that realize the retroreflective property of tapetum lucida have been reported [74]. For instance, three-dimensional photonic crystal structures have been demonstrated by self-assembling nanospheres in a silk hydrogel [75]. The poly(methylmethacrylate) (PMMA) nanospheres were stacked via self-assembly methods on the silicon substrate, and subsequently, the solution mixture of silk fibroin and stilbene chromophore was covered to fill out the PMMA opal structures and cured by a UV light source. By removing PMMA nanospheres embedded in the film, the silk hydrogel inverse opal (SHIO) was formed (Figure 4(b)), exhibiting high deformability. To verify the retroreflection properties of SHIO, a laser beam was projected on the half-ball agarose gel with SHIO, which presents higher retroreflection compared to the one without the SHIO (Figure 4(c)).

Interestingly, the elephantnose fish, *Gnathonemus petersii*, have adapted to improve vision in dim and turbid water through photonic crystal structures [23]. Unlike other animals that have evolved to increase overall photosensitivity or visual acuity, the retina of elephantnose fish features reflective photonic crystal cup structures, which do not act to increase photosensitivity nor high acuity. These peculiar retinal structures instead intensify specific wavelength (e.g., red) on the bottom of cups via light reflection from sidewalls on the cup (Figure 4(d)), coherent with the maximum absorption spectrum of cone cells. Thus, this red-sensitive retinal structure allows the elephantnose fish to improve vision in red-dominant turbid water. Inspired by the retinal cup structures of the elephantnose fish, Jiang’s group developed an artificial eye that can achieve high photosensitivity in low light environments [76]. The bioinspired photosensitivity enhancer (BPE) was fabricated through laser ablation processes to have a parabolic reflective sidewall design similar to the photonic crystal cups of elephantnose fish’s retina (Figure 4(e)). The BPE effectively enhanced light intensity under different light powers (Figure 4(f), green triangles), exhibiting better light collecting performance compared to the measurement without the BPE (Figure 4(f), blue squares [with BPE] and red circles [without BPE]).

Although human eyes are blind to polarized light, most arthropods and some vertebrates efficiently extract visual information from polarized light for sky navigation, communication, conspecific recognition, high contrast, and camouflage breaking. By utilizing polarization sensitivity, insects such as bees navigate by perceiving sky polarization pattern and some cephalopods communicate with their conspecifics and reveal the camouflage of prey [32], [33]. In the retina of polarization-sensitive animals, rod-like structures called microvilli are aligned in photoreceptors, transmitting or absorbing polarized light. For example, cuttlefish are sensitive to linearly polarized light, enabling them to enhance image contrast by filtering out polarized noises from light scattering underwater and to detect polarized light reflected from the fin of silvery fish [77], [78].

In this regard, a cuttlefish-eye-inspired camera that supports polarization vision for high contrast imaging was fabricated using carbon nanotubes (CNTs) polarization film [5]. The polymer film is stretched under heat to linearly aligned the embedded CNTs, so that the linearly aligned CNTs absorb or transmit linearly polarized light in perpendicular or parallel direction of the CNTs, respectively. The CNT polarization film effectively filtered linearly polarized light, implying potential application in high contrast imaging systems under extreme light conditions (e.g., polarized noise from light scattering or reflection), and also showed flexibility due to the soft nature of polymer film.

In particular, mantis shrimps are famous for their superior broadband spectral sensitivity as well as their polarization sensitivity, which allows them to detect both linearly and circularly polarized light [31], [79]. The ommatidia of mantis shrimps in 5th and 6th rows of the midband area have R8 rhabdoms, which consist of rod-like microvilli with nanoscale diameters. These microvilli show birefringence, allowing for the achromatic retardation of light. Thus, incident circularly polarized light undergoes quarter-wave retardation in R8 cells, converting to linearly polarized light, and the following R1–R7 cells analyze the polarization (Figure 4(g)).

By mimicking polarization vision in mantis shrimps, the polarization filter that can achieve full-Stokes polarimetric measurements was fabricated by adopting chiral metasurfaces [80]. As a configuration of tiered R8 and R1–7 cells, two layers were stacked with a dielectric spacer. The top layer, acting as R8 cells, was constructed by birefringent nanostructures, and the bottom layer consists of nanowire gratings for filtering linearly polarized light (Figure 4(h)). The optimized double-layer polarization filter presented high circular polarization extinction ratios of 35, exhibiting 80 % maximum transparency at NIR wavelengths. For implementing full-Stokes polarimetric measurements, four linear polarization filters with different orientations and two double-layer chiral filters with backup filters were integrated on a single chip. Along the eight random polarization states as inputs, the device successfully discriminated all states, as shown in Stokes parameters (*S*_0_–*S*_3_) (Figure 4(i)).

Although chiral metasurfaces can serve as circular polarization filters, optical structures inevitably induce a portion of light dissipation before it reaches the photoactive layer in the sensor, thereby increasing the burden in device miniaturization [81]. Recently, chiral materials such as chiral organic semiconductors, chiral conjugated polymers, inorganic materials, and chiral organic–inorganic hybrid perovskites have been explored to employ their absorption differences between left circularly polarization light (LCPL) and right circularly polarization light (RCPL) as a photoactive layer for photodetectors [82]. Li’s group demonstrated a circularly polarized light (CPL) photonic artificial synapse capable of conducting photodetection, learning, and recognition process [83]. The hybrid heterostructure of a helical chiral perovskite and single-wall carbon nanotubes enabled the detection of UV-CPL imaging and successfully imitated synaptic behaviors. Another group developed multi-wavelength (e.g., red [700 nm], green [556 nm], and blue [488 nm]) CPL photodetector based on an organic bilayer donor–acceptor heterojunction [84]. Due to the mechanical properties of organic materials, the fabricated photodetector not only detect CPL in multi-wavelengths, but also provide flexibility, making it one of the potential candidates for the visual component of artificial eyes inspired by mantis shrimp.

Together with polarization vision, it is noteworthy that the eyes of mantis shrimps provide 12 multiple spectral channels covering the wavelength range from 300 to 700 nm [85]. The spectral sensitivity of mantis shrimps is based on the penetration depths according to wavelengths. Recall from locations of R8 and R1–R7 cells, short wavelength light in the UV region is absorbed in R8 cells, while longer wavelengths are filtered by R1–R7 cells. In addition, crystal cones in each part of the eye also act as spectral filters, expanding spectral channels. Inspired by the multispectral vision of the mantis shrimp, a hexachromatic image sensor providing 6 spectral channels has been demonstrated for clinical applications [86]. To realize the multispectral properties on each pixel, three silicon photodiodes were stacked for absorbing photons with different wavelengths, and two bandpass filters (e.g., short-pass [<700 nm] and long-pass [>700 nm] filters) comprising dielectric multilayers were combined on each pixel to get a total of 6 spectral channels with vertically stacked photodiodes. The fabricated multispectral image sensor is advantageous for detecting fluorescent cancer tissues during surgery by highlighting fluorophores via NIR channels.

Likewise, many animals, including vertebrates (e.g., birds and fishes) and insects (e.g., bees and butterflies), have multispectral vision from UV to NIR wavelength regions. In the UV range, jumping spiders utilize their UV vision for foraging [87], conspecific communications [88], and mate choice [89]. Pollinating insects recognize patterns on flowers that can be revealed by UV detection [90], and birds can achieve high contrasts in vegetated environments enhanced by UV vision [91]. Inspired by the spectral vision of butterfly *Papilio xuthus*, a UV-sensitive CMOS image sensor has been demonstrated by vertically stacking photodiodes with a perovskite nanocrystal layer [92]. In the ommatidia of *P. xuthus*, a tiered structure of distal and proximal photorecpetors allows effective discrimination of different spectral wavelengths of light. The UV image sensor has been implemented based on the two photodetection strategies to detect both UVB spectrum (greater than 250 nm) and UVA spectrum (greater than 300 nm). The CsPbBr_3_ perovskite nanocrystal layer was deposited on top of the device to convert UVB light into visible fluorescence, while UVA spectrum is absorbed by the distal Si photodiodes. The bioinspired image sensor successfully conducted highly sensitive UV imaging for label-free biomarker detection, implying further applications in medical imaging and remote sensing.

Meanwhile, the detection of NIR and IR region exhibits quite different approaches. At the infrared region, animals sense IR radiation via thermosensory organ rather than eyes. Snakes use their pit membrane for IR detection and antennal tips of mosquitoes can detect IR sensing [93]. Several fishes and butterflies recognize NIR light through visual pigments of the eye [9]. For example, butterflies possess multispectral vision via photonic crystal structures in ommatidia, which consist of alternatively stacked layers of air and cytoplasm called tapetal filters (Figure 4(j)) [94], [95], [96]. The different stacks of tapetal filters in each ommatidium enable the recognition of a wide wavelength range of light. Inspired by the tapetal filters of *Morpho* butterflies, Gruev’s group demonstrated a monolithically integrated multispectral sensor (Figure 4(k)) [97]. The pixelated spectral filters were fabricated by stacking dielectric nanolayers of SiO_2_ and TiO_2_ on the customized CMOS sensor. The transmitted spectra are determined by optimizing the alternating stacks of nanolayers, which operates as an interference filter that transmits and reflects selective wavelengths of light. The fabricated tapetal filters deposited on the image sensor exhibited transmission of 60 % and 80 % at the visible and NIR regions, respectively (Figure 4(l)). In the clinical settings under surgical illumination, the multispectral imager collected both color and NIR fluorescence images, highlighting tumors in animal models and confirming its practical use for biomedical applications.

### 3.4 Retinomorphic devices inspired by animal eyes

To date, the major effort in bioinspired image sensors has focused on embodying the spherical geometry of retinas [11], [98], [99]. The curved retina in camera-type eyes endows economical designs in optics by compensating intrinsic optical aberrations like field curvature, enabling a single lens configuration in human and fish eyes. Various strategies have been adopted to realize curved image sensors for implementing artificial imaging systems capable of conducting similar visual features of animal eyes. Interconnection designs of metal electrodes including serpentine and fractal structures [7], [100], [101], origami and kirigami designs [102], [103], [104], and the direct growth techniques of perovskite materials [105] have embarked on a new era for artificial eyes. Several groups have developed bioinspired artificial eyes by deforming imagers into convex or concave forms, which enable simple optical designs [106], while also incorporating various optical features like zoom function or accommodation [107], and leads to the development of artificial compound eye via a convex framework [100]. Meanwhile, beyond the mimicking of the geometrical structures of retinas, functional features of signal transmission and processing performed from the retina to the brain have garnered recent attention in the field of bioinspired vision systems.

The images focused by the anterior eye optics are detected by the retina, which converts light signals into electrical signals. These signals are then sequentially transported in the form of pre-processed visual information to the visual cortex in the brain. Further elaborate processing takes place in the visual cortex to interpret, analyze them and make decisions for subsequent behaviors. In the artificial vision systems, image sensors act as a retina in biological eyes, but they can simply absorb photons and convert them into electrical currents. As a result, the back-end electronics of memory and computing units are required to support post-processing, including signal conversions from analog to digital and multiplexing, leading to a decrease in system efficiency in terms of slow speed, storage shortage, and an increase in hardware costs [11], [108]. However, rapid advancements in semiconductor and optoelectronics technologies have brought an unprecedented flood of visual information, demanding another breakthrough in imaging devices for high efficiency.

**Figure 5:**
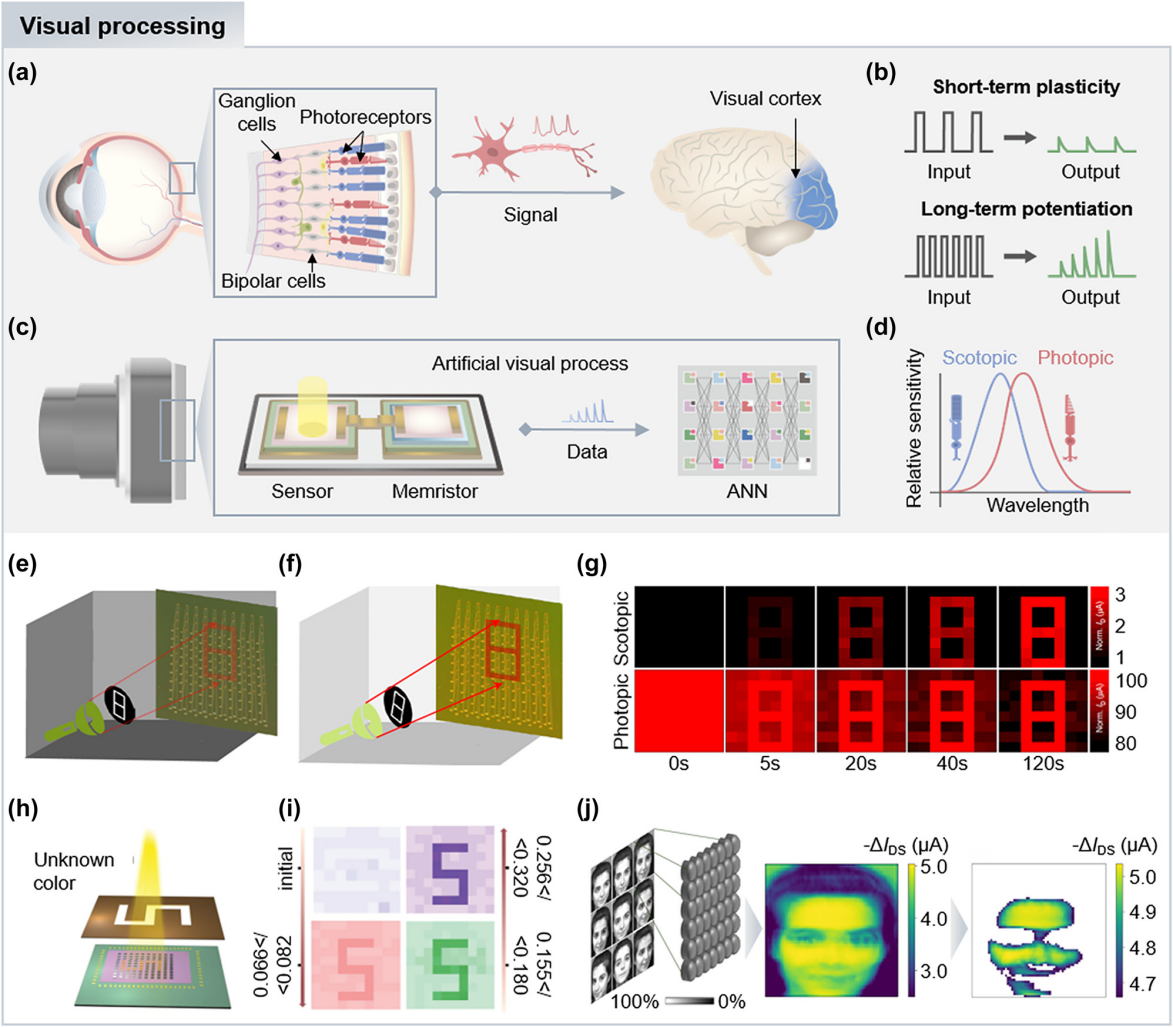
Bioinspired retinomorphic devices. (a) Visual process in human eyes. (b) Visual process in retinomorphic devices. (c) Schematics of input and output signals in short-term plasticity and long-term potentiation. (d) Relative sensitivity of cone and rod cells. The rod (cone) cells show a blue (red) shift in sensitivity, conducting scotopic (photopic) vision. Experimental illumination setups for confirming light adaptation of the MoS_2_ phototransistor under (e) dim and (f) bright background light conditions. (g) Scotopic and photopic adaptation results of the device, which exhibit increased image contrast over time. Reproduced with permission [115]. Copyright 2022, Springer Nature. (h) Schematic illustration of experimental setup for color discrimination test using light patterns with unknown colors. (i) Image results with color discrimination based on the different excitatory postsynaptic current ranges. Reproduced with permission [116]. Copyright 2022, Wiley-VCH GmbH. (j) Simulation process of the 64 by 64 array synaptic device for facial recognition. The trained recognition model consisting of a subset of synapses is constructed by training nine different facial images of a woman. Reproduced with permission [117]. Copyright 2023, Springer Nature.

Meanwhile, animals, including humans and insects with tiny brains, efficiently manipulate acquired visual information from the moment photoreceptor cells absorb photons to the signaling process via neurotransmission. For example, the visual process in human eyes is initially triggered by photon captures by photoreceptor cells (e.g., rod and cone cells). Subsequently, the light signals undergo pre-treatment with horizontal cells and transferred through bipolar cells (Figure 5(a)). These signals are further processed with bipolar, horizontal and amacrine cells, and are finally integrated by ganglion cells, which convey the resulting signals to the brain for advanced visual processing. The pre-processes of retinal cells are generated through parallel pathways, achieving a remarkably efficient pipeline. During the process, information delivery is accomplished by nerve cells (i.e., neurons) with synaptic plasticity (e.g., short-term plasticity [STP] and long-term potentiation [LTP]) (Figure 5(b)), enabling the operation of preliminary functions, including learning, recognition, and memorization by utilizing the synaptic weights.

To overcome challenges in conventional vision systems, retinomorphic devices (e.g., neuromorphic sensors and memristors) have been intensely explored to achieve highly efficient visual processing via in-sensor computing with artificial neural networks (Figure 5(c)) [109], [110], [111], [112]. For example, Kim’s group demonstrated a curved neuromorphic image sensor array based on the heterostructure of 2D and organic materials, mimicking human visual systems [113]. The charge trapping effects in the vertically stacked heterostructure of molybdenum disulfide (MOS_2_) and poly(1,3,5-trimethyl-1,3,5-trivinyl cyclotrisiloxane) enable quasi-linear time-dependent photocurrent generation and prolonged photocurrent decay, which exhibit responses similar to the neural plasticity of STP and LTP. These neuromorphic features of the sensor allow for facile noise reduction without additional electronics for memorizing and processing. In addition, the curved geometry of the sensor can collect images with simple optics (e.g., a single plano-convex lens), further alleviating hardware complexity induced by front optics.

The human eye adroitly optimizes its vision according to ambient light levels, with changes in sensitivity across the spectrum (Figure 5(d)) [114]. Under dark conditions, rod cells are primarily activated, with a blue shift in sensitivity, known as scotopic vision. In bright conditions, cone cells shift peak sensitivity to longer wavelengths, referred as photopic vision. It is noteworthy that the luminous efficiency of the scotopic curve is much higher than the photopic curve to attain sufficient image contrast under dim light.

Inspired by the light adaption of human vision systems, a MoS_2_ phototransistor array with time-dependent variation and inhibition characteristics has been demonstrated to enhance image contrast via scotopic and photopic adaptation [115]. For the scotopic adaption, negative gate voltages are applied under dim light to induce charge de-trapping in the band structure of the MoS_2_ transistor. This generates an increase in photocurrents over time, enhancing visual sensitivity. In contrast, positive gate voltages for photopic vision can increase charge trapping, resulting in a decrease in photocurrent over time and reduced sensitivity under bright conditions. To validate the light adaptation of the MoS_2_ phototransistor, imaging demonstration were conducted under dim and bright background light (Figure 5(e) and (f)). By applying positive (negative) gate voltages, the image contrast increased over time, demonstrating photopic (scotopic) adaptation (Figure 5(g)).

Shen’s group demonstrated a flexible artificial synaptic device for neuromorphic computing, encompassing memorization, learning, and color recognition [116]. The artificial synapse was fabricated using lead-free Cs_3_Bi_2_I_9_ nanocrystals to avoid the toxicities associated with metal halides, while still achieving optoelectronic synaptic behaviors. To emulate synaptic functions, charge trapping sites were intentionally induced by mismatching the bands of the Cs_3_Bi_2_I_9_ nanocrystals and the organic semiconducting layer. This feature allows programming of the device using light irradiation by utilizing trapped charges after irradiation, as well as an erasing process by applying a negative gate-source voltage. Interestingly, the device presented selective sensitivity to different wavelengths of light at 405, 532, and 635 nm, producing distinguishable excitatory postsynaptic current (EPSC) ranges (e.g., 588, 202, and 98 pA under light illumination at 405, 532, and 635 nm, respectively). Under irradiation with light patterns of unknown colors, such as red, green, and purple (Figure 5(h)), the resulting EPSC ranges were distinctive for different wavelengths (Figure 5(i)). The results indicate color recognition of the device, akin to color discrimination process in cone cells of the human retina.

Meanwhile, the organic electrochemical optoelectronic synapse has been demonstrated to overcome challenges associated with multilevel conductance states in traditional synaptic devices based on field-effect transistors [117]. These optoelectronic synapses were fabricated by combining donor-acceptor bulk-heterojunction interfaces as a photoactive layer in organic electrochemical transistors. The flows of anion and cation in the device occur photo-triggered electrochemical doping, which leads to generate electrical output signals, enabling synaptic plasticity. To validate synaptic behaviors similar to the human visual process, a 4 by 5 synaptic array was fabricated and successfully confirmed its image memorization by exhibiting a light pattern after exposure. Furthermore, researchers conducted simulations to assess practicality in facial recognition by simulating a 64 by 64 array synaptic device. The device was trained with nine different facial images of a woman, and during the process, a subset of synapses was constructed, forming a trained recognition model (Figure 5(j)). Subsequently, they established decision-making conditions for activating the device based on the received signals matching a trained facial model. In the facial recognition test, the simulated device effectively classified facial images and recognized the trained face of the identical woman based on the activation rates.

## 4 Conclusion and outlook

In this review, we discussed recent research on advanced visual components inspired by biological eyes to implement versatile visual processes for machines or robots. We have summarized the bioinspired surface structures to realize antireflective properties in nocturnal insect’s eyes, enhancing the transmittance of broadband and wide angle incident light. Beyond traditional circular apertures, various shapes in animal eyes have inspired the design of irregular-shaped pupils, expanding unique imaging features, including light-balancing ability. GRIN optics are also fascinating in lens design, enabling the reduction of the number of lenses and the use of complex surfaces. For dynamic imaging properties, tunable apertures and lenses have been demonstrated to implement light modulation and accommodation. Photonic structures in animal’s retinas exhibit diverse optical properties, enhancing light absorption and providing polarization/multispectral sensitivity. Retinomorphic devices have been intensely explored by emulating the retinal visual process in human eyes, including adaptation and recognition. Recent advances in bioinspired visual components have been accomplished with the development of nanofabrication and optoelectronic technologies; however, there are remaining gaps that need to be filled for practical applications.

The nanostructures are exposed on the surface of optical components, making them susceptible to destruction in harsh environments. Additional hard coatings can serve as a solution to enhance mechanical robustness, but further investigations are needed regarding their impact on the properties of AR nanostructures, particularly in terms of optical transmission and hydrophobicity [118]. Moreover, research on pupil shapes and tunable apertures is still in the rudimentary stage. While relatively simple shapes such as vertical and horizontal slit-like pupils have been simulated to explore their optical characteristics including depth estimation and panoramic view, respectively, other irregular-shaped pupils, such as crescent shapes of catfish, beaded shapes of geckos, heart shapes of toads, and other irregular shapes, are lack of studies on their visual advantages. Thus, additional research on optical design and implementation with shaped apertures is required. Commercially available GRIN optics mostly have rod shapes designed for laser collimation. Recent advancements in two-photon polymerization techniques could provide a means to fabricate GRIN lenses with various shapes. Metalenses, which can tailor optical wavefront, represent one strategy to realize various imaging properties observed in animal eyes. However, intrinsic challenges such as scaling up to centimeter sizes, increasing focusing efficacy, and achieving coverage across the entire visible region should be overcome for practical applications. Additionally, fabricating metalenses on curved substrate is highly desirable, as it may expand the potential for application in bioinspired imaging systems. In tunable optics, the slow response speed and high driving voltages are major considerations to be improved for practical applications. Compared to commercial image sensors providing resolutions in the hundreds of millions of pixels, curved image sensors in artificial eyes still have resolutions in hundreds of pixels. For evoking an industrial transition to flexible and curved image sensors, increasing pixel resolution is the foremost task. Simultaneously, the single pixel size of retinomorphic devices remains in the hundreds microns, necessitating further improvements. Also, beyond the laboratory-scale, photonic structures for light-trapping and polarization/multispectral sensitivity can be readily integrated on the imagers in large scale for their practical utilization.

Besides, there are numerous ocular structures exhibiting unique optical/photonic features waiting to be implemented for visual components. Avian eyes, for instance, possess a deep fovea in the central region of their retina, magnifying specific visual fields and enabling sensitive motion detection of target objects [119]. Deep-sea animals present interesting vision systems; for example, the spookfish can see a large field of view through the combination of front-view refractive optics and rear-view reflective optics [120], [121]. Magnetoreception is an intriguing subject that holds promise in unraveling how animals recognize Earth’s magnetic field and utilize it for navigation [122]. It is noteworthy that migratory songbirds have a protein in their photoreceptor cells, operating as a light-induced magnetic compass [123]. Additionally, researchers have recently discovered notable photonic structures in larval crustacean eyes that render them invisible against the background by reflecting different wavelengths from blue to yellow via ordering and size variation of nanospheres [124].

In conclusion, recent developments in bioinspired visual components provide various approaches to overcome challenges that arise in traditional camera systems. Together with ongoing research in biological eyes and optical/photonic fabrication techniques, it is expected that tailored visual components in artificial vision systems will enable future machines or robots to efficiently executing vision processes for specific occasions.
